# Evaluation of sexual dysfunction and its associated risk factors in the male partners of the infertile couples using International Index of Erectile Function

**DOI:** 10.4274/tjod.galenos.2019.89801

**Published:** 2020-04-06

**Authors:** Hajar Pasha, Mahbobeh Faramarzi, Zahra Basirat, Farzan Kheirkha, Hamid Shafee

**Affiliations:** 1 Babol University of Medical Sciences, Health Research Institute, Infertility and Reproductive Health Research Center, Babol, Iran; 2 Babol University of Medical Sciences, Social Determinants of Health Research Center, Babol, Iran; 3 Babol University of Medical Sciences, Department of Psychiatry, Babol, Iran; 4 Babol University of Medical Sciences, Department of Urology, Babol, Iran

**Keywords:** Infertility, male sexual dysfunction, risk factors for sexual dysfunction

## Abstract

**Objective:**

Sexual dysfunction is a major health concern in infertile men. This research aims to evaluate the sexual dysfunction and its associated risk factors in the male partners of infertile couples.

**Materials and Methods:**

The cross-sectional study was performed on 204 male partners of infertile couples that were referred to Fatemeh Zahra Infertility & Reproductive Center, Babol, Iran, in 2015. Sexual dysfunction was evaluated using The International Index of Erectile Function (IIEF). Logistic and linear regression tests were used for statis¬tical analyses. Statistical significance was considered with a p value less than 0.05.

**Results:**

The mean total IIEF score was 58.30±8.52. The lowest mean of IIEF domains was related to sexual desire and then orgasmic function in the male partners of the infertile couples. Erectile function contributed to the greatest amount of unique variance in the model for sexual function (p<0.001, R^2^=69.8%). The strongest correlation value was between the domains of overall satisfaction and intercourse satisfaction. There was a positive statistically significant association between sexual function with wife marital intimacy (p<0.002) and wife sexual function (p<0.001). There was a significant association between sexual dysfunction with job conditions (p<0.037, OR=0.094), and coitus count (p<0.009, OR=6.146). After adjusting for other variables, there was a significant association between sexual function and wife sexual function (p<0.005). Also, after adjusting for other variables, there was a significant association between sexual dysfunction and coitus count (p<0.004, OR=2.496), and job condition (p<0.046, OR=0.081).

**Conclusion:**

By considering sexual dysfunction and some related factors, early screening is required for distinguishing predictor factors of sexual dysfunction.

**PRECIS:** There were risk factors for sexual dysfunction in the male partners of the infertile couples and these able to affect sexual health.

## Introduction

Infertility is clinically described as the inability to conceive after 12 months of intercourse without using birth control^(1)^. Fertility problems can be observed in men and women in which the impaired fertility is experienced by 7-17% of couples; however, in over one-third of cases, the cause of infertility has been attributed to the men^(2,3)^. Infertility is a major crisis in the lives of infertile couples and associated with a heavy psychological burden, underlying the male sexual function^(4,5,6)^. Male sexual activity is an essential factor for the fertility^(7)^. There exist a reasonable connection between infertility and sexual disorder^(6)^. The sexual dysfunction can affect fertility and vice versa^(8)^. Several researches have examined the effect of infertility on the sexual experience^(9,10)^. It was found that the infertility might threaten the sexuality, competence, and male identity in infertile men^(10,11,12)^. In the infertile men, sexual dysfunction is an intricate issue^(13)^ that may have a deep impact on the quality of their sexual life^(14)^. Men with infertility experience a crisis that may have an injurious outcome on their sexual function with the perception of sexual inefficacy^(15)^. Many infertile men believe that infertility is associated with the loss of virility and masculinity, resulting in the sexual problems^(13)^. Since the sexual function is necessary for the reproduction^(7)^, identifying the sexual dysfunction and their risk factors are crucial in the infertile men before treatment of infertility^(16)^. Diagnosis of sexual disturbances in the male partners of the infertile couples can not only increase sexual function but also enhances natural pregnancy^(7)^. Many factors such as demographic conditions, poor relationships, biological and physical or emotional causes are known to be associated with the sexual dysfunction^(17,18,19)^. Moreover, the effect of cultural and social factors as well as the relationship between such aspects have not been well evaluated in the infertile men^(17)^, thus, more study will be required. Basically, careful assessments of male sexual dysfunction in understanding its detrimental consequences and in distinguishing its risk factors are important for prophylaxis efforts, which can be useful for counseling of the infertile partners.

To the best of our knowledge, there are a few studies in the literature on the infertile men to clarify the risk factors that shape the development of sexual dysfunction. However, more studies have been applied to establish the incidence rate of this topic in a healthy population. As sexual dysfunction is likely common in the infertile men, the evaluation of this matter is of the utmost importance. Therefore, this study aims to evaluate the sexual dysfunction along with the potential risk factor in Iranian infertile men.

## Materials and Methods

This cross-sectional study was done at Fatemeh Zahra Infertility and Reproductive Health Center of Babol Medical Sciences University, Iran in 2015. The duration of the present study was five months. Of the 220 eligible infertile men, 204 accepted to enroll in the project. This present study was conducted for the determination of sexual dysfunction and associated risk factors in the male partners of the infertile couples. All subjects were informed about the aims and details of the research and the secrecy of the data. Inclusion criteria were the ability of reading and writing, living with wife, history of >1 year of infertility, not having remarriage in couple, without any previous sterility, and not having a foster child. Exclusion criteria were major life events in the past months (death or difficult sickness in the family), currently using antidepressant and psychotropic drugs, physical and psychiatric problems, not having a stable sexual life for four previous weeks. The male partners of the infertile couples completed a demographic characteristics form, The International Index of Erectile Function (IIEF) questionnaire. The IIEF, which is an international index of erection function, was translated and validated into the Persian language. The IIEF Iranian version is valid and reliable for the Iranian population. The Cronbach’s alpha was from 0.73 to 0.99^(20,21)^. Its five domains includes sexual desire (2 items), erectile function (6 items), orgasmic function (2 items), intercourse satisfaction (3 items), and overall satisfaction (2 items). The score of each item ranges from 0/1 (no sexual dysfunction) to 5 (normal range). The range of subscale score including 2-10 for sexual desire; 1-30 for erection function, 0-10 for orgasmic function, 0-15 for intercourse satisfaction, and 2-10 for overall satisfaction. The total IIEF score is obtained from the sum of 15 items in the five domains that evaluate the sexual function. The range of the total score was 5-75. Lower values of this questionnaire represent worse sexual dysfunction. More studies have indicated its suitability to evaluate the sexual function in men. Also, in present study was used the instruments of Marital Intimacy Need Questionnaire and Female Sexual Function index for detecting of association between the male sexual function with the female marital intimacy and the female sexual function. These questionnaires were given to infertile women to be completed by their husbands.

The project with code number 1828 was approved by the Ethics Committee of the Babol University of Medical Sciences, Babol, Iran (approval number: 3326, date: 9/11/2013). Consent form was completed by all subjects.

### Statistical Analysis

Simple and multiple linear and logistic regression analysis models were estimated to calculate predictor factors for the sexual dysfunction among infertile men. Pearson’s correlation coefficient was used to determine the correlation between quantity data. All data analysis was conducted using SPSS, version 21 with p<0.05 indicating significance.

## Results

The mean age of male partners of the infertile couples and their wives was 31.77±5.47 (range from 20 to 50) and 27.82±5.70 (range from 17 to 43) years. The duration of marriage was 6.21±4.04 (range from 20 to 50) years. The residency in the majority of subjects was the private house (70.8%). The economic status of most the male partners of the infertile couples was moderate level (66%). The highest educational level in most of the subjects and their wives was high school diploma (33.8%, 40.1%). The mean and standard deviation of the duration of marriage was 6.21±4.04 (range from 2 to 20). The etiology of infertility in the majority of subjects was associated with male factors (37.1%). 92.1% of the infertile men had no child.

The mean and standard deviation of the IIEF was 58.30±8.52 (range from 27 to 75). The highest mean in IIEF domains is related to erectile function (23.27±4.26) (range from 9 to 30) and then intercourse satisfaction (10.70±2.42) (range from 0 to 15). The lowest mean in IIEF domains is related to sexual desire (7.75±1.52) (range from 4 to 14) and then orgasmic function (7.89±1.97) (range from 2 to 10). The mean of Overall satisfaction domain was (8.88±1.56) (range from 2 to 13). Standardized beta values showed that erectile function contributed to the greatest amount of unique variance to the model for infertile men sexual function (R^2^=69.8%), and followed by intercourse satisfaction (R^2^=54%), overall satisfaction (R^2^=41.3%), sexual desire (R^2^=39.6%), and orgasmic function (R^2^=31.8%) (p<0.001). The strongest correlation value was determined between overall satisfaction and intercourse satisfaction. There was a high correlation value between the domains of erectile function with intercourse satisfaction (Table 1).

A simple linear regression was done to assess the predictive nature of demographic characteristics on male sexual function. The results of the regression analysis are summarized in Table 2. There was not a significant association between the sexual function and age, wife’s age, the age difference of spouses, duration of the marriage, duration of infertility, and Body Mass index (BMI). There was a positive statistically significant association between the sexual function and wife’s marital intimacy (p<0.002) and wife’s sexual function (p<0.001). Furthermore, after adjusting other variables, there was a positive significant association between a sexual function with wife sexual function (p<0.05), and a trend towards a positive significant association between a sexual function with wife marital intimacy (p<0.57).

There was not a significant association between the sexual dysfunction with housing, economic status, wife’s educational level, current settlement type, educational level, infertility causes, treatment effort, infertility type, and previous using assisted reproductive technology (ART). There was a significant association between the sexual dysfunction with job conditions. The risk of sexual dysfunction was 0.094-fold less in employee than unemployed infertile men (p<0.037, OR=0.094). Also, there was a trend towards a significant association between the sexual dysfunction with the wife’s job. The risk of sexual dysfunction was 0.5-fold lower in infertile men when the job of their wives was employed than housewives (p<0.083, OR=0.500). There was a significant association between sexual dysfunction with Coitus Count when the frequency of coitus was monthly than >3 times/week, the risk of sexual dysfunction was 2-fold higher (p<0.009, OR=2.172). Also, when the frequency of coitus was 1-2 times/week than >3 times/week, the risk of sexual dysfunction was 6 -fold higher (p<0.009, OR=6.146). After adjusting other variables, there was a significant association between sexual dysfunction with job condition (p<0.046, OR=0.081), and coitus count (p<0.004, OR=2.496) (Table 3).

## Discussion

This study displayed the low average amount of IIEF among the male partners of the infertile couples. The low IIEF scores indicated more sexual dysfunction in subjects. A similar study showed that many male partners of the infertile couples reported decreased sexual function^(7)^. The average IIEF in the study of Moazeni-Bistgani and Mohammad-Alibeigi^(22) ^was similar with the our study, while the infertile men’s score was observed to be higher than other similar studies in total IIEF and also subdomains of sexual function such as sexual desire, orgasmic function, intercourse satisfaction, and overall satisfaction. According to a study (2015), the mean of total IIEF score was 45.7±7.5. Our study revealed a mean just higher than Turkish men diagnosed with infertility^(23)^. We believe that the difference in the average amount of IIEF and its subdomains of the infertile men in our protocol, compared with other studies, one reason is problems with the methodological issues and the other one cultural context.

Our findings suggest that the lowest average score in IIEF domains is related to sexual desire and then orgasmic function, which was nearly in line with the result of the study that it had done in Turkey^(23)^. A review of literature presented that in infertile men, hypoactive sexual desire and lack of sexual satisfaction were the most prevalent types of sexual dysfunction^(5)^. In another study, the average sexual desire and then orgasmic function scores were lower compared to other subscales of IIEF, which was similar to our study^(22)^. Also, Lotti and Maggi^(5)^ (2018) had reported that hypoactive sexual desire and sexual satisfaction were the most prevalent types of sexual dysfunctions in infertile men, while McCabe et al.^(24)^ (2016) showed that erectile disorder and premature ejaculation (Orgasmic disorders) were the most frequent sexual disorders in men.

The gathered data showed that in more than half of the cases, erectile function and then intercourse satisfaction contributed to the greatest amount of unique variance to the model for sexual function. A review of the literature indicated that the domains of sexual function such as arousal, and orgasm strongly were related to sexual satisfaction^(25)^. Another study showed that the men who experienced erection dysfunction had more negative expectations related to sexual function, and consider themselves as incompetent and weakly^(26)^. As erectile dysfunction is the most common sexual dysfunction in the male partners of the infertile couples, therefore; it can be considered as predicted sexual function to a considerable degree. It suggests that the various domains of sexual function are considered in sexual satisfaction and sexual function.

The results indicated that one of the most surprising results in our study was the strength of the correlation between overall satisfaction and intercourse satisfaction. Furthermore, there was a high correlation value between the domains of erectile function with intercourse satisfaction. The studies reported that more sexual satisfaction is related to the high frequency of sexual activity^(27)^. Several similar studies presented that more frequency of sexual function was associated with sexual satisfaction in men^(28,29)^.

In this study, there were some interesting results by analyzing the factors impressing sexual dysfunction. This allowed us to identify the risk factors of sexual dysfunction in the male partners of the infertile couples.

### Demographic Characteristics

Neither the age of men and women nor the age difference of spouses, duration of the marriage, duration of infertility, and BMI significantly contributed to the model for sexual function in infertile men. Also, there was not a significant association between the sexual dysfunction with housing, economic status, wife’s educational level, current settlement type, educational level, infertility causes, treatment effort, infertility type, and previous using ART. The study by Muller et al.^(30)^ demonstrated that sexual satisfaction was not associated with age, duration of the relationship, duration of treatment, and having a child in infertile men. We believe that the difference in significant association between a sexual function with some of the demographic characteristics in the infertile men in our protocol, compared with other studies, is caused by the variety of customs and cultures in our population. We think that the characteristics of the Iranian society may have an important role in the average amount of sexual dysfunction.

### Wife Sexual Function

The results of our study represented a positive association between a sexual function with wife sexual function. On the other hand, one of the key results in this study was that wife sexual function that was significantly related to sexual function for the male partners of the infertile couples. Favorable sexual function in the wife can be a sign of high sexual function in the husband. Alan et al.^(31)^ reported that female sexual function was a significant predictor of male partner sexual function. Female sexual performance can have positive effects on male sexual function. A similar study represented the interdependence of sexual satisfaction between partners, so that sexual complaints in husband often contributing to problems in sexual satisfaction or/and sexual function for wife and vice versa^(32)^. These results highlight the interaction between a female sexual function with male sexual function. The concept that the promotion of sexual function in the infertile men might be an important marker of female sexual health is emerging.

### Wife Marital Intimacy

The current study also showed that sexual function significantly was associated with wife marital intimacy. More wife marital intimacy was associated with improved sexual function in the infertile men. It is considered that sexual function is influenced by the marital intimacy of one’s partner. On the other hand; couple intimacy may alter sexual behavior. Studies presented that infertility is associated with the effects in couples and one partner’s response influences her or his partner’s response^(33)^. Theiss (2011) indicated that a lack of sexual intimacy was correlated with lower sexual satisfaction in married couples^(34)^. Findings from the same study showed that one of the psychological causes of sexual dysfunction is relationship or marital problems^(35)^. Emmanuel et al.^(36)^ reported that male sexual performance ability is vital in the marital relationship so that the lack of it can lead to a failure in the relationships. Basically, socio-cultural and interpersonal factors play an important role in developing sexual concern, which can lead to sexual difficulty and sexual dysfunction^(32)^. In our opinion, wife marital intimacy can consider proxies for sexual function in the male partners of the infertile couples.

### Job Condition/Wife Job

This present study shows that occupation is related to sexual dysfunction, as unemployed infertile men and women face complaints in sexual function more than employed individuals. The risk of sexual dysfunction in employed men was 0.094-fold less compared to unemployed men. Also, there was a trend towards a significant association between the sexual dysfunction with the wife’s job. The risk of sexual dysfunction was 0.5-fold less when the occupation of the infertile men’s wives was an employee than a housekeeper. Sexual performance in men and women undergoing infertility is positively affected by job conditions. Principally, one of the barriers to sexual satisfaction can be a lack of occupational support. The review of the literature showed that social factors such as occupation can influence the sexual dysfunction^(29)^. Pasha et al.^(37)^ showed that there was poor marital intimacy in husbands who were unemployed than those with job. Basically, favorable job, appropriated social status, and good financial situation can lead to improved sexual and marital satisfaction. Unemployment and not having occupation may have an important effect on marital satisfaction^(38,39)^. Also, for both infertile men and women, the job can consider the most common source of financial support, which is needed for paying heavy costs of infertility treatment. High cost of infertility treatment can lead to persistence concerns. In fact, having a job increases the chance that individuals can pay the cost of infertility treatment. On the other hand, financial support and occupation are essential for having desirable sexual performance. Alirezaie et al.^(40)^ indicated that income and high costs of infertility treatment have an influence on sexual function. Low-income individuals have a sexual complaint 4 times more compared with high-income individuals. To confirm this statement, Audu^(41)^ found the income effect on sexual function. Low income is a risk factor for sexual disturbance. Difficulty in paying costs of infertility treatment raised the chance of sexual problem up to nine times^(42)^. The authors offer two possible explanations for this result: At first, it seems that job is one of the important factors that a person is able to cover the costs of infertility treatment. Second, the lower socioeconomic situation is also associated with less physical and mental health, which can be linked with the sexual dysfunction.

### Coitus Count

Gathered data from the present study found that the sexual dysfunction was higher among infertile men who had a low frequency of intercourse. The rate of sexual dysfunction was 2-fold higher when the frequency of coitus was monthly than >3 times/week. The results of a study on the subject showed that the quality of couples’ relationships may influence the sexual dysfunction^(29)^. Data from a similar study revealed that more frequency of sexual activity can lead to high sexual satisfaction^(27)^. These data are important because they will determine whether appropriate intercourse frequency can be suggested to these infertile couples to improve sexual function and fertility. Perlis N et al.^(43)^ in the study as “Coital frequency and infertility: which male factors predict less frequent coitus among the infertile couple?” reported that the infertile men with better erectile function had 1-12 times more frequent coitus. Erectile dysfunction can be considered as a risk factor for less frequent intercourse. Therefore, coital frequency should be assessed in infertility protocol.

### Strengths and Weaknesses

The present study has several strengths and weaknesses. We used a validated, internationally established questionnaire IIEF to assess the sexual dysfunction. Providing the correct answer to question about sexual issues was a limitation of the research, which was somewhat reduced by giving confidence to patients about the confidentiality of information. It is not possible to consider whether the study subjects representatives of the male partners of the infertile couples in general.

## Conclusion

From the data obtained in the male partners of the infertile couples, we can observe that sexual dysfunction is common in unemployed men and their housewives, wife’s sexual dysfunction, poor wife’s marital intimacy, and monthly intercourse. Therefore, our findings strongly suggest the routine clinical investigation of risk factors for sexual function in the male partners of the infertile couples. Good understanding of the risk factors of sexual dysfunction is essential to assay male infertility and sexual complaints.

## Figures and Tables

**Table 1:**
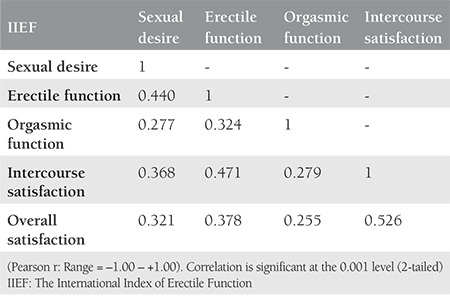
The International Index of Erectile Function domains intercorrelations

**Table 2:**
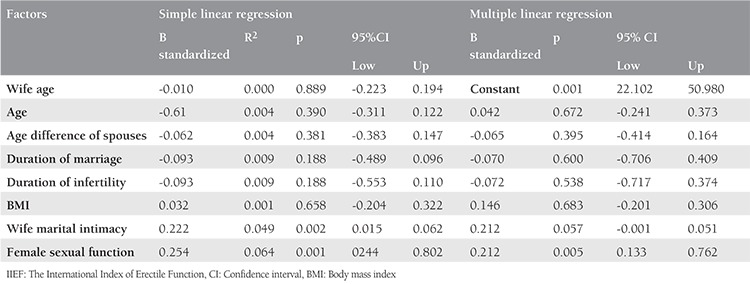
Simple and multiple linear regression analysis of the International Index of Erectile Function with the other variables in the male partners of the infertile couples

**Table 3:**
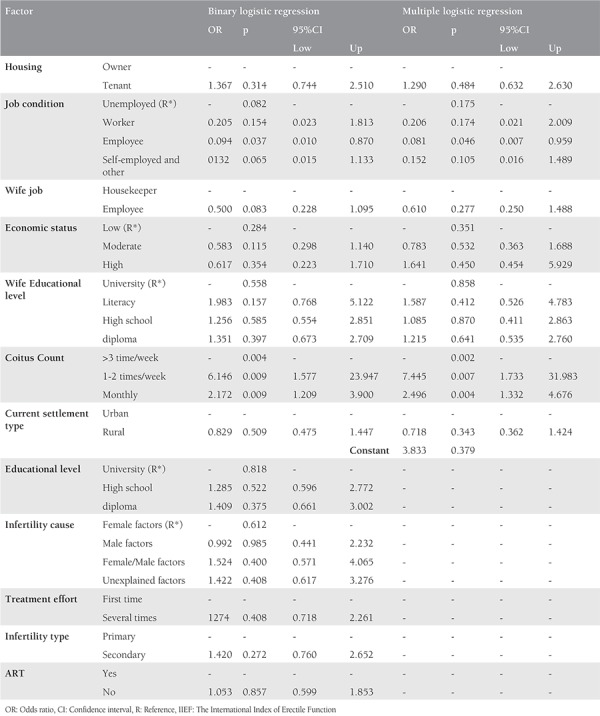
Binary and multiple logistic regression analysis of IIEF with the other variables in the male partners of the infertile couples
